# Sanitizing the national body: COVID-19 and the revival of Japan’s “Closed Country” strategy

**DOI:** 10.1177/01171968221125482

**Published:** 2022-09

**Authors:** Gabriele Vogt, Sian Qin

**Affiliations:** 9183 Ludwig Maximilian University of Munich

**Keywords:** Japan, COVID-19 pandemic, *jishuku*, border-closure, international migration, pandemic othering, authoritarian populism, performance of crisis

## Abstract

Japan’s handling of border control measures during the COVID-19 pandemic has become known as *sakoku*-approach. *Sakoku* literally means “closed country” and generally refers to a historic period when the Tokugawa Shogunate (1603–1868) kept Japan’s borders shut because international contacts were feared to cause public upheaval and political instability. While these times have long passed, contemporary Japan, too, is known for its tight management of immigration avenues. In the light of the COVID-19 pandemic, most of these avenues were cut off, and despite much criticism, have remained largely inaccessible for two years now. In this paper, we build on concepts from authoritarian populism and the performance of crisis to analyze how and why Japan revived its isolationist strategy. We decipher the discursive framings that Prime Minister Abe applied to illustrate the disruptive influence that open borders would have on Japan’s public health, social stability and by extension, on the national body itself. We argue that from the onset of the pandemic on, the ethnic others were portrayed as a risk mainly for two intertwined reasons: Firstly, Japan’s pandemic management relies on self-constraint rather than rules and sanctions, and the ethnic others’ compliance was not fully trusted. Secondly, this exclusionary strategy fed into populist discourses and was presumed to result in favorable support rates for the administration.

## Introduction

In early 2020, when the COVID-19 pandemic reached the shores of Japan, the country quickly closed its borders to any international travel of foreigners. Not only did tourism and short-term business trips come to an abrupt halt, but also foreign residents of Japan, in some cases, found themselves stranded overseas as Japan banned them from returning home while the international travel of Japanese citizens was not restricted at any point (Burgess, 2021; Korekawa, 2021). Meanwhile, the foreign residents of Japan have again been granted the same travel privileges as Japanese citizens. However, even when considering the two exceptional groups that, during the pandemic, moved through the “Olympic/Paralympic travel bubble”^1^ and the short-lived framework of the “Business/Residence track,“^2^ Japan, to this day, by and large remains a closed country to international arrivals (Dujarric, 2022; Lee and Inuma, 2022; Takahashi, 2022). In stark contrast, *within* Japan mobility has never been limited during the pandemic. Residents have never had to deal with curfews or restrictions on outings. The government has even initiated and subsidized a domestic travel campaign to boost the tourism industry which has been hit hard by the absence of international travelers (Asahi Shimbun, 2021; Go To Toraberu, n.d.). This means that the international travel ban was and is not supposed to limit non-necessary travel to and in Japan altogether, but is much more designed to terminate the influx of foreigners – be they mere travelers, future or even current residents – while, in Japan, life largely went on without any severe limitations.

This paper studies Japan’s “closed country” strategy which resembles the nation’s historic *sakoku* (“closed country”) period of 250 years that ended only in the mid-19th century.^3^ Historically, this strategy was implemented to protect Japan from Western influences such as Christianity and fire weapons, both of which had been made responsible for previously causing social upheavals in Japan. As we have seen in more recent times, e.g., in Japan’s foreigner crime discourse in the 2000s (Yamamoto, 2004), in times of crisis, Japanese policymakers tend to follow a strategy of segregating the “foreign” (the “outsiders”) from the “Japanese” (the “insiders”) to fend off real or perceived danger. We may understand this recurring strategy as an effort to ensure a sanitized, i.e., healthy and orderly national body of Japan by creating and enforcing boundaries to foreign influences in the form of transborder human mobility. In this paper, we detangle how and why the current closed-door strategy has become a core element in Japan’s countermeasures against the COVID-19 pandemic. We show how Japan has followed a strategy of “pandemic othering” (Foster, 2021: 46) and exclusion of foreigners as a core element of its approach to sanitize the national body in times of a global health crisis. Based on a qualitative content analysis of long-term Prime Minister Shinzō Abe’s speeches in 2020, we address the discursive framings used to portray foreigners in the early months of the pandemic which established the “closed country” strategy that Abe’s two successors so far have followed suit.

## Research design

We address the puzzle of Japan’s “closed country” strategy with two hypotheses: Firstly, we argue that the current border-closures are a case of an exclusionary policy that reflects Japan’s previous approaches to immigration and integration, and in very practical means, the current strategy is also utilized to counterbalance Japan’s toothless domestic pandemic management. Secondly, we focus on the political dimension inherent in the strategy of closed borders and argue that Prime Minister Abe deliberately used this populist strategy to bolster his dwindling support rates. Thus, maintaining an isolationist strategy amidst much international criticism, was and is supposed to, firstly, protect the public health, the social stability and the wellbeing of the national body of Japan; and secondly, to strengthen the political power of Prime Minister Abe and the ruling Liberal Democratic Party (LDP).

### Theoretical background

Conceptually, we center our study at the intersection of authoritarian populism and the performance of crisis when addressing the phenomenon of a discursive and practical securitization of international migration by Prime Minister Abe. We understand both populism and crises as dynamic processes and agree with the scholarly literature pointing out that oftentimes, crises are constructed and performed to serve populist strategies (Moffitt and Tormey, 2014; Norris and Inglehart, 2019; Taggart, 2004). As Taggart writes, “populism tends to emerge when there is a strong *sense* of crisis and populists use that sense to inject an urgency and an importance to their message” (2004: 275). Undoubtedly, the COVID-19 pandemic must be understood as a global health crisis as the “objective notion of crisis itself” (Moffitt, 2015: 195). The political choice of how to address a crisis, however, depends on whether people also develop a “*sense* of crisis” (Taggart, 2004: 275). Next to the objectifiable dimension, a crisis, then, also inherently holds a constructivist dimension: A crisis becomes “a phenomenon that is mediated and performed, and experienced culturally and socially” (Moffitt, 2015: 195).

The performance of crisis is a multi-step process that includes creating, shaping and prolonging an objectifiable (or a constructed) crisis for political purposes. Authoritarian populism, which is conducted by governmental actors, strives for a hegemony of norms that are diffused in a top-down manner, e.g., through educational campaigns aimed to interfere with everyday life actions (Hall, 1988; Moffitt, 2015; Takeda, 2021). To an authoritarian populist, the aim of performing a crisis is to (re-)define and strengthen norms or ideologies that are inherent in a specific cultural or social setting. Not only is a hegemonic norm bound to displace other norms, but it will eventually also oust other more pragmatic interests, such as economic ones, which it might clash with (Takeda, 2021). This happens, e.g., when cultural threats connected to immigration supersede the economic interest to recruit foreign workers. Anti-immigration attitudes are often associated with authoritarian populist values and strategies (Norris and Inglehart, 2019), and the securitization of immigration becomes a core element in the performance of crisis. One central characteristic of populist strategies is to pitch “us” (“insiders”) versus “them” (“outsiders”). The single marker of ethnicity is used to define these groups and to subscribe to the “outsiders” some profound distrust. This discursive framing, then, constitutes the reasoning for segregationist policies directed at non-nationals. Borrowing the words of Norris and Inglehart (2019: 205): “Authoritarianism is about protecting ‘Us’ from ‘Them’ – those who transgress community norms.”

In our study on the border-closure policy directed at foreign nationals during the COVID-19 pandemic, we show how discursive framings and political output manifested foreigners as those “outside” the newly emerging hegemonic norm of how the Japanese were supposed to act during the pandemic. Under the leadership of Prime Minister Abe, foreigners were discursively constructed as those who would pose a risk to Japan’s national body and domestic sanitization efforts, and therefore, had to be kept at the shore and prevented from entering Japan. In line with Moffitt, who suggests that “the performance of crisis should be seen as an essential core feature of populism itself” (Moffitt, 2015: 211), we understand this process of crisis performance as an act of authoritarian populism. With our analysis, we contribute a case study to enhance the awareness and understanding of populism in Japan, and add to the emerging literature that counters the assumption that Japan, compared to many other liberal democracies, seems almost “‘immune’ to the rise of populist demagogues” (Fahey et al., 2022: 2).

### Data

As data for our analysis, we used the manuscripts of speeches and press conferences of Prime Minister Abe in three decisive moments of the pandemic management in its early months when the closed-border strategy was manifested. Even after Abe stepped down on 16 September 2020, his successors in office, both from the same political party, the Liberal Democratic Party of Japan (LDP), Yoshihide Suga (until 29 September 2021) and Fumio Kishida (as of present), have largely remained steadfast to the closed-border strategy. We focus our analysis, firstly, on the handling of the COVID-19 outbreak on the Diamond Princess cruise ship which was quarantined in the harbor of Yokohama City in February 2020. Secondly, on the implementation and lifting of a state of emergency in April and May 2020 respectively; and thirdly, on the kick-off of a governmental travel campaign in July 2020. We provide a qualitative content analysis of the Prime Minister’s core speeches on all three occasions and decipher discursive narrations of segregation of the foreign “outsiders” from the Japanese “insiders” used to justify an ongoing securitization of immigration in Japanese politics.

### Authoritarian populism and the performance of crisis in Japan’s border-closure policy

During the COVID-19 pandemic, Japan has managed to keep both infection numbers and fatality cases low. By the summer of 2020, i.e., six months into the pandemic and marking the closing point of our analysis in this paper, fewer than 30,000 infection cases and under 1,000 deaths from COVID-19 had been counted for Japan (Slater, 2020). Japan’s routine of mask wearing, greeting rituals without bodily contact, people’s low levels of obesity and diabetes, and their experience with handling natural disasters have been mentioned as potential reasons for Japan’s “pandemic miracle” (Aldrich and Yoshida 2020: 217). That Japan has been conducting a remarkably low number of PCR tests and thus, many infections may have gone unnoticed and unregistered, is another factor that partially explains the low numbers. While the debate is ongoing (Aldrich and Yoshida, 2020; Iwasaki and Grubaugh, 2020; Suppasri et al., 2021; Wright, 2021), some would put it in a nutshell by saying that “Japan seems to have lucked out for reasons we may never fully understand” (Slater, 2020: 1).

Several social scientists have come to agree that in lieu of any coherent pandemic policy, a top-down activation of Japan’s culture of self-restraint (*jishuku*) – in this case refraining from any unnecessary activities – is at the center of Japan’s comparably low infection numbers and fatality cases. Nakano (2021) argues that Japan has, in fact, no other policy in place to counteract the spread of COVID-19 than to request individuals to act upon self-restraint. Burgess (2021: 8) adds that the government’s approach was matched with substantial social control, i.e., while self-restraint was “ostensibly ‘voluntary’ there was strong peer-pressure to isolate for the ‘common good’.” Soon, in fact, the term *jishuku keisatsu* (self-restraint police) emerged, which hinted to citizens’ social control of fellow citizens’ behaviors and denunciation of misbehavior, quite in the character of a “para-police by mutual monitoring” (Iijima, 2021: 294). Aldrich and Yoshida (2020: 220) even claim that “if anything has saved Japan from the pandemic, it has been the people themselves.” In other words, citizens have been widely mobilized under a “seemingly apolitical invented tradition of compliance” (Wright, 2021: 468). Wright (2021: 468) continues to argue that the culture of self-restraint “can be deployed as a tool to shape citizen behaviors through the mobilization and construction of a metanarrative of imagined homogenous ethnonational identity.” *Jishuku* needs to be understood as a hegemonic norm that juxtaposes “us” fellow citizens with “them,” i.e., the foreigners who are positioned outside this imagined community and therefore willingly or unwillingly may “transgress [the] community norm(s)” (Norris and Inglehart, 2019: 205) of self-restraint.

In the following subsections, we analyze how the implementation of the hegemonic norm of self-restraint has been implemented in an intertwined process with border-closure measures during the first months of Japan’s pandemic management. To this end, we study the discursive framings of Prime Minister Abe in three decisive phases of this process. The Diamond Princess cruise ship quarantine marks the onset of Japan’s policy to keep foreigners at bay. With the first state of emergency, the norm of self-restraint was implemented and discursively made hegemonic; and with the campaign “Go To Travel,” the “new normal” (*nyū nōmaru*) way of living with this norm in isolationist Japan was consolidated domestically.

### The Diamond Princess cruise ship

The Diamond Princess cruise ship has been docked at Yokohama Port since 3 February 2020. Two days prior, it had been confirmed that one passenger who had previously disembarked the ship in Hong Kong tested positive to COVID-19. Upon the ship’s arrival in Yokohama, the Japanese Ministry of Health, Labour and Welfare (MHLW) decided to deny, all 2,666 passengers (1,281 of whom were Japanese nationals) and the 1,045 crew members from 56 different countries, disembarking privileges; instead, they were required to quarantine on board. COVID-19 spread on the ship, and by 23 February 2020, when passengers were finally granted permission to disembark, the number of confirmed infection cases had risen to 691, and two persons had died of the virus (Asahi Shimbun, 2020b; Nakazawa et al., 2020).^4^

The COVID-19 infection and fatality numbers aboard the Diamond Princess cruise ship have continuously been reported separately from Japan’s numbers (Adelstein and Yamamoto, 2020; Maly, 2020). A similar separated count was used to refer to infection cases detected in Japanese citizens undergoing COVID-19 tests at airport immigration counters, thereby establishing and manifesting the “sharp distinction between the off-shore pandemic and safety inside the country” (Maly, 2020).^5^ This reporting pattern is a first indicator of Japan’s *mizugiwa taisaku* (measures to secure the shore) approach to become a dominant policy. Foreign influence, particularly if feared to be troublesome, was to be kept at the shore of the Japanese territory – be it on a cruise ship. The same strategy was applied during the actual *sakoku* (“closed country”) period, when a small number of foreigners were allowed to reside for trading and educational purposes on Dejima Island, an artificially constructed island in Nagasaki Bay, and therefore not on Japanese soil (Helleiner, 2021; Palmer, 2016).

After a meeting of the National Security Council, Japan’s Ministry of Land, Infrastructure, Transport and Tourism (MLIT) on grounds of the Immigration Control and Refugee Recognition Act, moved to refuse passengers on board another cruise ship (MS Westerdam) that was scheduled to dock at Naha Port in the prefecture of Okinawa, permission to disembark (Nakazawa et al., 2020). Of the 1,455 passengers aboard this ship, four were Japanese citizens; of the 802 crew members, one was Japanese (Mainichi Shimbun, 2020). In this situation, at a press conference on 6 February 2020, Prime Minister Abe announced that as of midnight, Japan would move to deny entry to foreigners (*gaikokujin no nyūkoku wo kyohi suru*) (Cabinet Secretariat, 2020a). Using the terminology of *mizugiwa taisaku* (measures to secure the shore), the prime minister pledged to thoroughly follow through with border-closure measures. He also pledged to enhance domestic surveillance measures to prevent the spread of infections, and repeatedly stressed that his administration was acting quickly and firmly in this unprecedented situation (Cabinet Secretariat, 2020a).

The terminology of *mizugiwa taisaku* has previously been used in the context of border enforcement and delivers the unmistakable notion of crime prevention (Yamamoto, 2004). In a large-scale initiative spearheaded by the Ministry of Justice (MOJ) from 2004 onwards, undocumented foreigners in Japan, whose number had then peaked at 220,000 persons, were to be identified and deported. As part of the campaign, the National Police Agency (NPA) ran posters that showed one presumably foreign man surrounded and tackled by several police officers. The wording on the poster read “*mizugiwa de fusegu, mamoru*” (stop them at the shore and protect [the nation]) (Vogt, 2014: 57) and called on the Japanese citizens to support authorities in their mission against undocumented foreigners. To the Japanese public, Abe’s choice of terminology in his speech must have rung the bell of the foreigner crime discourses of the early 2000s.

Shortly after the prime minister’s statement, the Mainichi Shimbun (2020), a major daily newspaper in Japan, ran a piece revealing that Minister Kazuyoshi Akabane (MLIT), apparently, called on ships with route to Japan to voluntarily refrain from docking in Japan. He coined this the “self-restraint from attempting domestic disembarking” (*kokunai nyūkō jishuku*), which indicates, next to “securing the shores,” the second approach in Japan’s pandemic management, i.e., calling for self-restraint. This may have laid the tone to a fruitless debate among cabinet members who had raised the question whether it would constitute a violation of human rights (*jinken shingai*) if also Japanese citizens aboard the MS Westerdam were denied entry to Japan (Asahi Shimbun, 2020d). No mention apparently was made regarding any rights of foreign citizens in this meeting behind closed doors. According to the Asahi Shimbun (2020d), a senior government official suggested to sidestep any controversial public debates about this topic by simply calling the passengers “foreign nationals aboard the cruise ship Westerdam from Hong Kong” (*Hong Kong hatsu senpaku uesuterudamu ni jōsen shite iru gaikokujin*).

After Abe initially had announced to stop international arrivals to Japan, a move with which he aimed to present “simple solutions and strong leadership” (Moffitt, 2015: 198), Japan’s border-closures proceeded in steps depending on the place of departure of foreign travelers arriving from certain provinces of China, and later South Korea, before 1 April 2020. The borders were closed to travelers from 49 countries and the list of banned destinations continued to grow further over the following weeks. In this timeline of border closures, we see, on the one hand, some consideration regarding an objectifiable risk which is connected to the travelers’ country of departure, the infection numbers there, and by extension, the likelihood that travelers may have already contracted the virus. On the other hand, however, the performative dimension of crisis became apparent in the (overly) simple classification of border-closures directed at foreigners but not at Japanese. In fact, Japanese nationals were being repatriated from the COVID-19 epicenter in Wuhan, China, and were asked to undergo COVID-19 testing upon arrival and to self-isolate for 14 days. However, neither testing nor self-isolation had been made mandatory and the government simply asked for citizens’ voluntary compliance. Two of the repatriated Japanese, however, refused compliance, and the prime minister reacted to their denial simply by “bemoan[ing] how ‘extremely regrettable’ the situation had become” (De Vries, 2020).

While even deviant behavior among Japanese was grudgingly accepted, a strict border-closure policy limiting all foreigners from entering Japan – including foreigners whose residence was in Japan – was in place until the end of August 2020. The only exception applied to special permanent residents, i.e., those who reside in Japan as descendants of former colonial subjects, the so-called *zainichi*, many of whom are of Korean and Taiwanese ancestry (Burgess, 2021). This exclusionary policy, based solely on the grounds of citizenship, made members of Japan’s foreign community feel like “they were being treated like second-class citizens” (Burgess, 2021: 8) and left “a ‘scar’” (Burgess, 2021: 9) on them. At the time of writing, in spring 2022, some limited entry options for workers and students are being discussed in the administration of current Prime Minister Fumio Kishida (Asahi Shimbun, 2022). Despite facing rising international criticism, e.g., by business representatives,^6^ students and academics,^7^ and by the World Health Organization (WHO) who claims that closing borders to international travelers but not to citizens lacks medical reasoning (Miki, 2022; Johnston, 2022; Yokoyama, 2022), the Japanese government continues to hold onto the basic narrative of its closed-border strategy, i.e., that the pandemic is mainly a risk being imported to Japan from international travelers.^8^

### The first state of emergency

In spring 2020, infection numbers were on the rise in Japan and it became clear to policy-makers that border closures alone, which remained in effect unreversed, would not prevent the spread of COVID-19 in Japan. The virus had started circulating in Japan. Moreover, significant regional differences concerning the spread of the disease were apparent, with densely populated areas such as metropolitan Tokyo and popular tourism spots, which had experienced much inflow also of domestic travelers due to winter festivals, most heavily impacted (Tokyo Shimbun, 2020). The pressure on the Abe administration to act and to implement measures that could counter the domestic spread of the disease was rising (Suzuki, 2020; Nishida, 2020).

However, Prime Minister Abe’s public statement on how to proceed did not come before 7 April 2020. In the meantime, some of the heavily impacted prefectures had already moved forward independently: Naomichi Suzuki, the Governor of Hokkaido on 28 February 2020, and Yuriko Koike, the Governor of Tokyo on 23 March 2020, directly addressed citizens and asked for their cooperation in combatting the spread of the virus. In the absence of any legal basis for lockdown-like measures, both governors requested citizens to voluntarily employ self-restraint (*jishuku*). Both addressed their speech to the residents of the various territories, i.e., to *dōmin* in the case of Hokkaido and to the *tomin* of Tokyo. By using this terminology, no distinction was made regarding the citizenship of those addressed, and we may very well understand that foreign residents were included in this appeal.^9^ In Hokkaido, citizens were initially urged to follow *gaishutsu jishuku* (self-restraining from outings) for two days; in Tokyo, the request was to follow *ibento jishuku* (self-restraining from events) (Hokkaido Government, 2020; Tokyo Metropolitan Government, 2020). These are the first two mentions by government representatives of *jishuku* as a citizen-based countermeasure to the pandemic. Given the infection numbers in both prefectures, the daringness of Suzuki, Japan’s youngest prefectural governor at the time, and the unyieldingness of political veteran Koike, it may not come as a surprise that it was them who set the populist tone for the core domestic measure to address the pandemic, i.e., citizen cooperation.

Prime Minister Abe on 7 April 2020, finally addressed the nation in a speech, claiming that the medical system was facing mounting pressures to attend to all COVID-19 patients. He declared that this development had prompted the government to declare a state of emergency in Tokyo and six prefectures (Saitama, Chiba, Kanagawa, Osaka, Hyōgo and Fukuoka),^10^ based on Article 32 of the Act on Special Measures (*tokubetsu sochi-hō*). According to the Prime Minister, to protect the public health and the wellbeing of the national body of Japan, everybody would now need to do their part: most importantly, people needed to change their behavior (*nani yori mo kokumin no minasama no kōdō henyo*). He continued: for the next month until the end of the Golden Week holiday season, we all need to reduce our contacts by 70 to 80 percent; he culminated this request in a strong “please do not go out” (*gaishutsu jishuku wo onegai itashimasu*). Also, he repeatedly stressed that Japan’s approach to a containment of the virus was completely different from the scenes that were seen in other countries and referred to examples from other G7 nations (which Japanese politicians generally like to use as reference point). No lockdown measures or roadblocks around certain regions^11^ were to be seen in Japan if citizen compliance was determined enough. The prime minister ended his speech by drawing a parallel to the hardships that the nation faced after the 11 March 2011 Great East Japan Earthquake. He also pointed out that what gave hope to many people then were the strong interpersonal bonds (*kizuna*) that they experienced, and implied that if this spirit was revived now, Japan would be able to overcome this national crisis (*kokkateki na kiki*) the pandemic was causing (Cabinet Secretariat, 2020b).

In his address to the nation, Abe appealed to people’s emotions. He called on them to unite and to find hope and confidence in the strong interpersonal relations that are deemed to be a part of Japan’s communitarian value set. He also several times juxtaposed this Japanese approach of COVID-19 management to the approach of other countries, predominantly based on rules and sanctions, which he painted in somewhat gruesome pictures. He thereby added to his emotional appeal some indirect warning regarding potential alternatives to the voluntary measures his administration has decided on. Drawing on people’s mobility data from major train stations in Tokyo, we can conclude that his appeal to reduce mobility resonated with the people. The number of passengers frequenting, e.g., Shinjuku station, the crossing point of major inner-city train lines dropped by 70 percent immediately after the state of emergency was declared (Nippon Television Network Corporation, n.d.; Takahashi, 2020).

However, when the prime minister attempted to portray his own self-restraint in a somewhat inept tweet including a video clip on 12 April 2020, he was heavily criticized. The video showed himself in house slippers as he played with his dog, sipped a cup of tea, etc., while all along being serenaded by famous singer-songwriter, Gen Hoshino. While this could have been taken as humoristic, since the video was paired with another urgent message much in the style of his address to the nation,^12^ the tweet was received with bewilderment. The prime minister was depicted as bored, trying hard to “do nothing” while the nation’s medical workers were overwhelmed, and many citizens were struggling with pandemic life to an existential degree (Abe, 2020). Here, the prime minister clearly was not in tune with people’s emotions. This tweet may be one major reason, also next to the distribution of two washable cloth masks (so-called “Abenomask”) to every household in Japan,^13^ which manifested the public image of the prime minister’s rather weak leadership qualities presented during the pandemic. The portrayal of his policy initiatives in the media ranged from “respectful disagreement to acerbic parody” (Wright, 2021: 460), and the support rates of his administration kept falling (Iijima, 2021). While the support rate of the Abe administration stood at 45 percent at the onset of the pandemic in February 2020, it had dropped to 43 percent in March 2020 and to 39 percent in April 2020. It remained unchanged from this low level for three months (NHK, n.d.).

Yet, in response to the dwindling support rates, Abe neither changed actual policies nor framings used to describe and to justify these policies. Quite on the contrary, he continued to apply a strategy of performing a crisis and prolonging citizens’ *sense* of crisis, as can be seen in his speech on 14 May 2020, when he announced to extend the state of emergency, and even in his speech on 25 May 2020, when he announced to lift the state of emergency. On 14 May 2020, he claimed that efforts by everyone were more crucial than ever (*kore made ijō ni ohitori ohitori no gokyōryoku ga hitsuyō*) (Cabinet Secretariat, 2020c). However, mobility data of Shinjuku station shows that people have regained some of their mobility, which means that the willingness to continuously act upon self-restraint has been waning (Nippon Television Network Corporation, n. d.). On 25 May 2020, the prime minister eventually announced that the state of emergency was to be lifted. Nevertheless, he called for people’s compliance and efforts to continue the new lifestyle (*atarashii seikatsuyōshiki wo korekara mo*) of social distancing and mask wearing. Only by creating a new normal in everyday lives (*aratana nichijō wo tsukuriageru*) would the nation be able to prevent the worst (*saiaku no jitai*), i.e., infection numbers spiraling out of control as it was seen in other parts of the world (Cabinet Secretariat, 2020d).

While Abe continued to point to other countries as setting bad examples in COVID-19 management and claimed this to be the reason for the need to continue banning foreigners from entering Japan, the foreign community *within* Japan, to some degree, also kept experiencing an exclusionary and discriminatory attitude. In May 2020, disinformation spread on social media claiming that foreigners made up more than half of the infection cases registered in Japan (Burgess, 2021). Also, in later months of the pandemic, rumors like this kept emerging, e.g., in November 2020, when an infection cluster in a Brazilian ethnic school prompted social media posts encouraging parents “not to let their children play with ‘foreign’ kids” (Burgess, 2021: 8), or in May 2021, when a health center in Ibaraki prefecture “warned people ‘not to eat with foreigners’” (Burgess, 2021: 8). In the small town of Beppu, in Oita Prefecture, where Ritsumeikan Asia Pacific University, the country’s university with the highest share of international students is located, foreign students started experiencing verbal abuse and hands-on discrimination; some local restaurants or hair salons would no longer serve them (Kyodo News, 2020). This “Japanese Only” attitude of shop owners, sadly, is not new to Japan, as Debito (2004) has previously shown for a hot spring resort in Hokkaido. Yamanaka (2003) has covered the famous case of Ana Bortz, a Brazilian resident of Shizuoka Prefecture, who had been denied entry to a jewelry shop on the grounds of her being a foreigner.

The exclusionary attitude toward foreigners during the COVID-19 pandemic may have reached a hitherto unprecedented level when not only shop owners, but also members of government joined the discriminatory chorus. Most famously, when Tarō Aso, then Deputy Prime Minister and Finance Minister, claimed that the secret to Japan’s comparatively successful COVID-19 management is its *mindo* (level of the people).^14^ On 4 June 2020, in an Upper House Finance Committee meeting, Aso reported of frequent phone calls he would get from his G7 counterparts asking why Japan’s COVID-19 fatality rate was much lower than theirs. Apparently in the meeting, he said, “*I told these people, ‘Between your country and our country, mindo (the level of people) is different’. And that made them speechless and quiet. Every time.*” (Asahi Shimbun, 2020a). As was pointed out by Kim (2020), the term *mindo* was frequently used during colonial times, in particular, when Japan would assess the so-called *mindo* of the Korean peninsula. The term would then refer to people’s educational level and hygienic practices, but also their crime rate, standard of living, ability to use technology and even the tax potential of a region. When Aso praised the high level of Japanese people’s *mindo* – deliberately using this term “with its connotations of ethno-nationalist superiority” (Wright, 2021: 465) – and juxtaposed this with everybody else’s *mindo*, he implied that foreigners posed a threat to the governmental efforts of sanitizing the national body of Japan from the virus; after all, non-Japanese could not be trusted to live up to Japanese standards.

We may conclude that while foreigners, on first sight, received equal treatment in Japan during the pandemic – this includes access to the initial JPY 100,000 fixed payment (*tokubetsu teigaku kyūfukin*) granted to all residents as a compensation for experienced economic hardships, and later to free vaccination shots (Burgess, 2021) – there have been several incidents in which business owners or even politicians expressed a stance that reveals how they think foreigners in Japan are not and probably cannot be integrated into society as full members; as “insiders” in the tightly knit web of the communitarian values that also *jishuku* (self-restraint) is based on. In more practical terms, at the onset of the pandemic, migrant workers oftentimes were the first to be laid off and experienced some severe worsening of their living conditions (Tran, 2020). Thus, on a discursive and practical level, foreigners experienced pandemic othering in the ethno-nationalist society of Japan (Foster, 2021; Liu-Farrer, 2020).

### The “Go To Travel” campaign

The “Go To Travel” (*Go To Toraberu*) campaign, which kicked off on 22 July 2020, the day the Tokyo Olympic Games had originally been scheduled to commence, marks the transition into practicing the new lifestyle (*atarashii seikatsuyōshiki*) that Prime Minister Abe called for in his 25 May 2020 speech when he announced the end of the state of emergency. The governmental campaign offered heavy discounts on overnight stays at hotels and included a “cash back” coupon system which was supposed to stimulate consumption at the destination, thereby providing a multifaceted economic help package to tourist spots, many of which struggled enormously amidst the lack of international travelers (Korekawa, 2021; Johnston, 2020). All residents of Japan, including the foreign residents, were eligible to take part in the campaign (JTB, 2020).

In a speech on 20 April 2020, Abe advertised the “Go To Travel” campaign as of a large enough scale to compensate some of the economic losses that were expected due to the postponement of the Tokyo Olympic and Paralympic Games by one year. He pledged to stimulate consumption by increasing the domestic flow of people (*kokunai no hito no nagare*). The prime minister’s framing restores a somewhat natural taste to these travel activities, which during the heydays of the *jishuku*-time would have been deemed utterly un-solidaric. In fact, during the months of the campaign period which lasted from 22 July to 28 December 2020, self-restraint has been promulgated occasionally by different governmental agencies, but no other state of emergency has been declared by the national government. This newly induced mobility, however, was tightly interwoven with a set of new manners regarding social distancing and mask wearing, which were propagated as educational campaigns throughout the country by posters and flyers designed in the Cabinet Office of Japan (Cabinet Secretariat, n. d.).

From a viewpoint of infectious disease control, a governmental campaign inducing people’s mobility around the country seems counterintuitive; and indeed, the campaign is now associated with rising infection numbers throughout Japan that summer (Anzai and Nishiura, 2021).^15^ This, it seems, was the price the Abe administration was willing to pay for three outcomes that they aspired for with the campaign: firstly, to support the hospitality industry which was hit hard by the lack of international tourists, and thereby, to dampen any potential calls for reopening the borders to international travelers from some of the big businesses of the industry, such as the airlines;^16^ secondly, to eventually restart international tourism to Japan; and thirdly, before leaving the isolationist path of closed-borders, to consolidate the newly established norms of a *jishuku*-lifestyle with masks, social distancing and new manners among the Japanese public and the country’s foreign community who were included in this educational effort. We can thus understand the “Go To Travel” campaign as a first step toward ascribing the meaning of “new normal” to the lifestyle of self-restraint, and to ensuring that this new hegemonic norm would spread widely and firmly throughout the country (including its foreign community) and remain steadfast even amidst the eventual opening of the borders to international travelers (Matsuyama, 2022).

## Discussion

In this paper, we set out to detangle through a qualitative content analysis of Prime Minister Abe’s major speeches in spring 2020, how and why Japan’s closed-door strategy has become a core element in the country’s countermeasures against the COVID-19 pandemic. We have seen in the reactions to the cruise ship infection cluster, in the enactment of a state of emergency, and in the counter-intuitive travel campaign, that Japan’s approach to COVID-19 management was relatively soft when directed at the inside of the nation, and rigid when distancing that nation from physical interaction with the outside world. To this day, two years into the pandemic, the nation’s borders by and large remain shut, and inside of Japan, COVID-19 measures continue to rely on the self-restraint applied by citizens and foreign residents. While this self-restraint has started to undergo a redefinition from “not doing” to “doing it in a different manner,” the core element of this approach remains the “solidaristic behavior of imagined fellow citizens” (Wright, 2021: 453). Foreign residents in Japan, to some degree, i.e., when it comes to diffusing new hegemonic norms through educational campaigns, had to be included as a target group. This was necessary to ensure that measures designed to contain the virus and by extension, to sanitize the national body of Japan, were successful. With regard to border crossing travel privileges, however, foreign residents during the onset of the pandemic clearly were not included in the category of “imagined fellow citizens” (Wright, 2021: 453), but remained “second-class citizens” (Burgess, 2021: 8). To this day, nonresident foreigners can enter Japan only to a very limited degree. This treatment reflects a (re)production of a new pattern of “semi-citizenship,” as proposed by Morris-Suzuki, in which “greater rights for a particular ‘minority’,” i.e., international mobility mainly owned by foreign migrants crossing the Japanese border, “are seen as eroding or devaluing the rights of the ‘majority’” (Morris-Suzuki, 2015: 82), i.e., mainly Japanese citizens staying within the safety and secured space inside Japan.

Politically, Japan’s “closed country” strategy serves as a case study of authoritarian populism that did not translate into rising support rates for the government. Prime Minister Abe and his administration used border closures as a core element of performing a crisis on top of an objectifiable global health crisis. While this strategy may have contributed to Japan’s success in keeping the COVID-19 infection cases and fatality rates low compared to other G7 nations (Naito, 2021), the strategy proved unsuccessful in other ways. Most importantly, it did not induce any rally around the flag effect (Foster, 2021) which can often be observed when a *sense* of crisis is eminent.^17^ Quite on the contrary, though, in the first and second quarter of 2020, the Prime Minister’s support rates fell to a degree that he eventually had to resign after eight years in office. Only after the implementation of the “Go To Travel” campaign, a heavy economic stimulus package, did the cabinet’s approval rate rise. In July 2020, it stood at 44 percent (the same as in March 2020) (NHK, n.d.). We cannot see, however, any boost to his approval rates as a result of border-closures, and one possible explanation for this might be the “‘battle of flags’” (Kato and Yoshimoto, 2020: 29) effect. Meaning, rather than the Prime Minister with his symbolic exclusionary policy and occasional gaffes, it was local figures such as prefectural governors whose hands-on handling of the pandemic successfully managed to transfer the crisis perception into political capital.

In line with Triandafyllidou’s (2022: 6) observation that the “pandemic and related international border restrictions have emphasized the existence of different layers of membership within each country” that distinguish “citizens from residents from aliens,” we argue that this process has indeed been apparent in Japan. Every administration since the onset of the pandemic has made clear in narrative and practice that full membership in the society was withheld from foreign residents. However, for the sake of keeping the nation’s body sanitized and healthy, they, too, were expected to comply with the same norms as Japanese citizens. Moreover, while admired by many around the globe for its cultural capital and economic attractiveness, based on the narrative of securing the sanitized body of the Japanese nation from potentially harmful foreign influence, these administrations have decided, in an act of authoritarian populism, to close the borders to international migration to Japan. One media report pointedly evaluated Japan’s exclusionary approach as Japan being on its way from “cool Japan” to “cruel Japan” (Adelstein, 2022). Accordingly, as a medium-to long-term effect of this exclusionary layering, the revival of Japan’s “closed country” strategy is likely to result in the nation’s relative loss in soft power and international standing. However, given its dire economic situation and quickly progressing demographic aging of its domestic workforce, Japan cannot afford this loss. Quite on the contrary, Japan needs to regain its appeal among an international workforce willing to take up jobs in the high, medium and low skilled segments.^18^ Preventing its most recent “closed country” period from backfiring on the nation’s economy will be one of the most pressing tasks for Japan’s policymakers in the post-pandemic future.

## Notes

1 This refers to the special conditions that were applied to athletes, coaches and officials affiliated with the Olympic and Paralympic Games that were postponed by one year and eventually held in Tokyo in summer 2021. Those in the “bubble” faced a strict regime of frequent PCR testing and restrictions regarding interactions with persons outside the “bubble.” In sum, about 54,250 international arrivals were considered the “Olympic/Paralympic travel bubble” during the period of 1 July through 6 September 2021 (Olympic and Paralympic Games Tokyo 2020 Coordination Division, 2021).

2 The “Business/Residence track” was initially designed to allow “talents necessary for doing business” to cross Japan’s borders; the business track aimed at short-term business travelers and the residence track at employees dispatched to Japan by their international employers for a longer period (Ministry of Foreign Affairs of Japan, 2022). These two tracks were approved by the Japanese government on 18 June 2020, i.e., toward the end of Prime Minister Shinzō Abe’s administration, and implemented on a minimal scale from August 2020 until his resignation in September 2020. Both schemes were expanded to also include “students, families, and spouses” during the early months of the administration of Prime Minister Yoshihide Suga, Abe’s successor, before being suspended in January 2021 amidst the global spread of a new virus variant (Immigration Services Agency of Japan, 2021). For instance, from August 2020 to January 2021, 63,484 technical interns have come to Japan under this track (Tokyo Shimbun, 2021). With 43.2 percent, they constitute the largest target group of this migration avenue, followed by international students (28.9 percent or 42,531 persons). Both groups contribute heavily to the workforce in the low skilled segment, and the opening of the “Business/Residence track” may be considered a concession of policymakers to the demands for particularly low-wage laborers that is continuously voiced by business representatives that include Masakazu Tokura, Chairman of the Japan Business Federation (Keidanren Japan Business Federation, 2022).

3 See the collection of articles on “Reiwa no *sakoku*” (“closed country” of Reiwa period i.e., current emperor’s period of reign), which one of Japan’s major daily newspapers, the Mainichi Shimbun, has compiled (Mainichi Shimbun, n.d.).

4 Kentaro Iwata, one of Japan’s leading experts on infectious diseases who had been called to serve on board the ship as the situation worsened, described the chaotic quarantine protocol that he witnessed first-handedly in YouTube videos, which circulated widely in Japan (Wright, 2021).

5 Adelstein and Yamamoto (2020) point out that “the cruise ship on the port of Yokohama is the second largest epicenter of the virus in the world, right after Wuhan. But since the cruise ship is not a country, it generally hasn’t been described that way.” We may add that, while “the cruise ship is not a country,” Japan’s main goal regarding the cruise ship outbreak was to ensure that this “epicenter of the virus” was under no circumstances associated with the infection dynamic in Japan either.

6 This includes the chairpersons of the Tokyo offices of several long-standing international business associations, such as the American Chamber of Commerce and the European Business Council. Also, Masakazu Tokura, chairperson of the Japan Business Federation (Keidanren), is quoted saying that “it was ‘unrealistic’ for the government to ban the entry of foreign nationals” (Johnston, 2022) any longer.

7 Many of them have been mobilizing on Twitter under hashtags like, e.g., #educationisnottourism.

8 Prime Minister Fumio Kishida justified Japan’s restrictive policy when claiming that the *mizugiwa taisaku* approach proved a success, and that Japan, under his leadership – other than other G7 nations – was not going to risk any of this success by throwing caution overboard now (Cabinet Secretariat, 2022).

9 Several prefectures as well as national-level ministries provided material on governmental appeals and policies in several languages; again, indicating that efforts were being made to include foreign residents in this strategy of self-responsibility.

10 The state of emergency was expanded to all 47 prefectures of Japan on 16 April 2020 (Anzai and Nishiura, 2021).

11 At that time, e.g., in Italy, some 50,000 people were confined in their cities behind roadblocks to forcefully reduce people’s mobility, and by extension, the spread of the virus (Adelstein and Yamamoto, 2020).

12 The message read: “We cannot meet our friends. We cannot meet in a bar and have a drink. However, when we all act like this, we will save many lives. And, if we all now endure these hardships with much rigor, we will alleviate the challenges our medical workers face. I pray with all my heart for the compliance of every single one.” (Abe, 2020)

13 “Abenomask” was a pun on the “Abenomics” economic strategy. The masks were extremely small and hardly covered the mouth and nose of an adult. Many of them were also substandard in their production, yet, the distribution of the masks amounted to an enormous JPY 46.6 billion (Wright, 2021).

14 Wright (2021: 465) translates this as: “‘cultural level.’“

15 Already when the campaign kicked off, infection numbers in various regions of Japan had been on the rise again, e.g., in Tokyo and Okinawa. Tokyo was officially excluded from the initial phase of the campaign (Cabinet Secretariat, 2020e), and in the prefecture of Okinawa, Governor Denny Tamaki declared a prefecture-wide state of emergency amidst rising infection numbers and asked travelers not to come to Okinawa (Asahi Shimbun, 2020c; Anzai and Nishiura, 2021).

16 Indeed, early on, criticism of the border-closure policy was voiced by business representatives from the hospitality industry and the educational sector, such as universities and Japanese language schools (NHK, 2022).

17 It is even more surprising that the prime minister’s exclusionary or anti-immigrant stance did not translate into rising support numbers, when considering that in Japan, as elsewhere, pro-immigration policies are correlated with unfavorable voter perceptions (Strausz, 2019) and we might expect a vice versa effect.

18 The Japan International Cooperation Agency (JICA) estimates that 6.74 million, or four times as many migrant workers than there are today, will be needed by 2040 to sustain the growth of the Japanese economy at current levels (Japan International Cooperation Agency, 2022).
